# A statistical physics view of swarming bacteria

**DOI:** 10.1186/s40462-019-0153-9

**Published:** 2019-03-15

**Authors:** Avraham Be’er, Gil Ariel

**Affiliations:** 1 Zuckerberg Institute for Water Research, The Jacob Blaustein Institutes for Desert Research, Ben-Gurion University of the Negev, Sede Boqer Campus, 84990 Midreshet Ben-Gurion, Israel; 2 Department of Physics, Ben-Gurion University of the Negev, 84105 Beer Sheva, Israel; 3 Department of Mathematics, Bar-Ilan University, 52000 Ramat Gan, Israel

## Abstract

Bacterial swarming is a collective mode of motion in which cells migrate rapidly over surfaces, forming dynamic patterns of whirls and jets. This review presents a physical point of view of swarming bacteria, with an emphasis on the statistical properties of the swarm dynamics as observed in experiments. The basic physical principles underlying the swarm and their relation to contemporary theories of collective motion and active matter are reviewed and discussed in the context of the biological properties of swarming cells. We suggest a paradigm according to which bacteria have optimized some of their physical properties as a strategy for rapid surface translocation. In other words, cells take advantage of favorable physics, enabling efficient expansion that enhances survival under harsh conditions.

## Introduction

Swarming typically refers to the natural phenomenon of many organisms or agents performing some group movement, such as the synchronized migration of cancer cells, aggregation of insects, flocking or schooling behavior of birds and fish, human crowds and more [37, 51, 123, 126, 132]. In the context of bacteria, the term swarming usually refers to *a specific type* of motion in which rod-shaped flagellated bacteria migrate rapidly on surfaces en masse [17, 40, 71, 75, 93]. By saying “a specific type”, we mean that swarming is a particular biological mode that some bacterial species can transition into. This transition involves several cellular processes such as changes in the expression of key proteins, in chemical communications between bacteria as well as mechanical changes [60, 71, 72, 74, 129, 144]. For example, bacteria can alter the aspect ratio of their cells and grow extra flagella prior to swarming [60, 71, 72, 74, 129, 144]. Therefore, despite many similarities, swarming is not just collective movement (e.g., swimming) at high densities (e.g., [34, 119, 121]). These subtleties can be important, in particular because swarming is a natural state, i.e., cells collectively “decide” to transition into swarming (compared to dense swimming which is typically studied in artificially concentrated suspensions [120]). This suggests that the changes in cells prior to the onset of swarming may bear advantage to the colony’s survival. Swarming is typically characterized by densely packed clusters of bacteria moving in coherent swirling patterns of whirls and flows that can persist for several seconds [6, 11, 12, 19, 33, 40, 68, 142, 143]. In addition, unlike bacteria that swim in bulk, swarming bacteria are in a constant interaction with a surface boundary [10].

From a physical point of view, bacterial swarms are a biological example of active matter [43, 80]. Active particles take in and use energy to generate motion or self-propulsion [104]. In swarming bacteria, movement is achieved by rotation of the flagella. The contemporary viewpoint of active systems as a kind of material that can be studied using the tools of non-equilibrium statistical physics has brought forth deep understanding of the universal properties of active systems and showed a wealth of new phenomena [83, 104]. This review focuses on understanding and analyzing the fundamental dynamical aspects of this fascinating natural phase of active matter called bacterial swarming as studied in laboratory experiments.

A relevant and called upon question is why focus on swarming bacteria and not, for example, on a more general scope such as collectively swimming micro-organisms or self-propelled rods? In this review, we promote the idea that bacterial swarming offers a unique opportunity for studying the tight coupling between the biological aspects of bacterial colonies and the physical aspects of these systems. Particular emphasis is given to explore the connection between the mechanical properties underlying cell motion through the medium and the statistical properties of the collective. For example, changing the shape of the cell is a complicated bio-mechanical process which requires valuable energy and resources [67]. The reason that cells invest in these processes, even at harsh conditions when resources are scarce, suggests it may bear some advantage to the survival success of the organism. Indeed, as we will show below, these mechanical changes can affect the statistics of the swarm dynamics and, as a result, impact the swarm ability to colonize new territories and face stress, including increased resistance to antibiotics [19, 25, 76, 78, 92, 106]. Accordingly, the main paradigm we propose is that bacteria manipulate their cellular properties and the environment to promote favorable physics with advantageous dynamical properties.

The biochemistry of swarming bacteria has been extensively researched in the micro-biological literature [40, 60, 61, 71, 74, 75, 94, 129]. It has been shown that swarming involves particular regulation of gene expressions related to a wide range of cellular processes, such as chemo-sensory mechanisms, synthesis and assembly of flagella, depression of cell division and more [60, 71]. For this reason, the definition of bacterial swarming as flagellated (surface) motion has been challenged. Kaiser [71] suggests that, due to many biochemical similarities, other surface translocation methods such as pulling (using pili), pushing (over secreted slime) and gliding, for example in aggregation of myxobacteria [72, 144], should also be considered as swarming. However, here, we adopt the former approach and concentrate on motion generated using flagella.

There has also been considerable recent progress in understanding the physics underlying the swarming phenomenon, including the physical interactions between cells [19, 65], interactions with the medium [10, 11, 73], and the statistical properties of the swarm (e.g., [107, 142, 143]). Similar works of collective swimming bacteria are also of relevance [32, 34, 45, 46, 82, 108, 119–121, 137]. This review attempts to present a broad account of the fundamental biological and physical principles underlying bacterial swarms, with an emphasis on the relations between the two. As a result, some aspects are only reviewed in brief. Additional details can be found in the literature referred to. We begin with a short introduction to the relevant biophysical background (“Biophysical background” section). Section “Statistical properties of the swarm dynamics” is the core of this review, which details the major statistical properties of the swarm dynamics as observed in experiments, and their analysis using the tools of statistical physics. It is divided into two subsections, relating to two types of swarms: monolayer and multilayer swarms. Although both types have some common elements, mono- and multi-layer swarms are prepared differently in the lab and therefore constitute different biological manifestations of swarms. Comparing the two types sheds light on the impact the biological setup has on the swarm dynamics. Section “Theoretical aspects” shifts to the physicist point-of-view, in which bacteria are viewed as a statistical ensemble of particles with appropriate properties. The section briefly reviews some of the relevant theoretical results from the rapidly growing fields of collective motion and active matter. The implications of these theories to bacterial swarms are discussed. In section “An individual within the crowd”, we address the interesting problem of studying what an individual cell actually does within a swarm. Additional swarming related phenomena, including swarming bacterial species which were not discussed in previous sections, are surveyed in section “Further swarming related phenomena”. We conclude with our personal view on interesting open directions for future research.

## Biophysical background

**Fig. 1:**
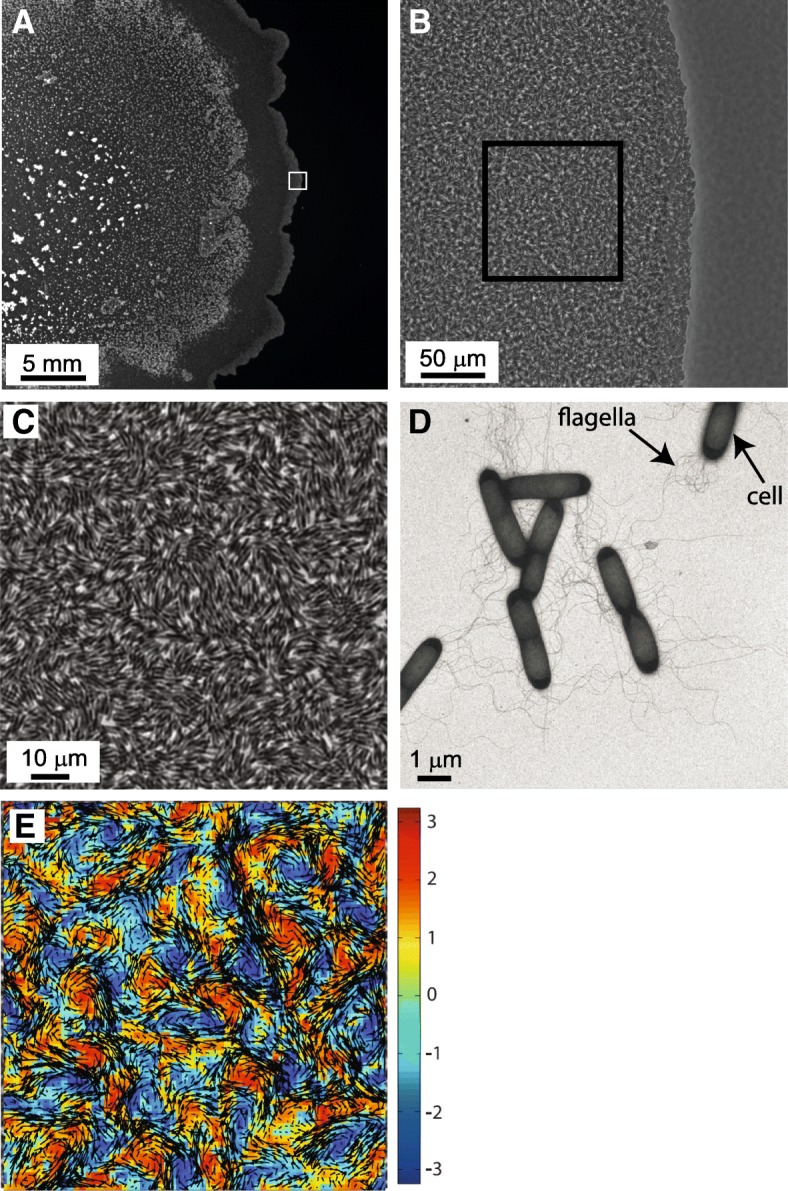
The swarming colony – a multiscale view. **a** A macroscopic top-view of a swarm colony grown on an agar plate, indicating the region where microscopic analysis is performed (the size of the frame is a bit larger than the entire field shown in (**b**)). **b** A microscopic view of the colony; cells are nearly resolved in the multilayered structure. Frame indicates the region where optical flow measurements are performed. **c** The highest magnification of the colony (taken using a 60X objective lens), **d** TEM images of bacteria taken from the swarm. Multiple flagella are visible. **e** The velocity and vorticity fields of the region in (**c**). Black arrows indicate instantaneous (and local) velocity, and colors indicate vorticity (scale bar in rad/sec)

The swarm - the active part of a bacterial colony undergoing swarming, where the flagellated cells move rapidly, traps a water reservoir. In this moist region, individual cell speed is comparable to swimming speeds in bulk liquid, typically of the order of 20 μm/sec [40, 93, 134]; See Figs. 1a-d for a typical experimental setup. The continuously circling motion of individual bacteria within an expanding swarm is apparently random, undirected and independent of the chemotactic signaling system [7, 93, 116]. This is in contrast to swimming bacteria, which migrate towards a nutrient source using a biased random walk controlled by chemosensory signal transduction or chemotaxis [16]. Figure 1e shows the approximated flow field for a swarm using an optical flow algorithm [103]. A large variety of bacterial species are able to swarm in the lab, yielding similar patterns, which demonstrates the phenomenological robustness of this phenomenon. Examples for swarming species are *Escherichia coli* [38, 40, 128], *Bacillus subtilis* [10, 75, 142], *Serratia marcescens* [5], *Salmonella* [17, 94, 124], *Paenibacillus dendritiformis* [11] *P*. *vortex* [6, 130], *Proteus mirabilis* [129] and *Pseudomonas aeruginosa* [90], all were studied intensively in the lab by different groups. In-situ swarming studies (e.g., in-vivo, or on medical equipment) are rare. One known example, which may be of medical importance, is swarming of *P. mirabilis* in catheter-associated urinary tract infection, were the swarming cells are attached firmly to the medical equipment surfaces and migrate from the urethral meatus into the bladder [70].

Swarming may be largely divided into two categories according to the thickness of the swarm. (1) *Monolayer* swarms, which usually involve species that secrete large amounts of surfactants, for example *B. subtilis*. In this regime, the advancing colonial edge is relatively sparse, where the bacteria migrate rapidly, forming only a thin, single layer of moving cells. Towards the interior of the colony, cells become more crowded, swirl much faster, but still occupy only a monolayer. Further back inside the colony, cells become less active and pile (in some cases they exhibit sporulation or biofilm formation). (2) *Multilayer* swarms (e.g., in the case of *S*. *marcescens*) are much more crowded at the advancing edge, forming a thick layer of cells (usually in the range of 2–10 layers), with an even thicker swarm structure (order of 100’s of active layers) in the interior.

The physical mechanisms that play a role during swarming, as well as the characteristics of the swarming patterns, are known to depend on both the cell characteristics and the environmental conditions. The first includes cell density [142], cell aspect ratio [65] and cell rigidity [14, 62], flagellar density [129], flagellar number and structure [36, 55] and flagellar propulsion power and activity [58, 128], interactions between flagella of adjutant cells [38] and the ability to secrete biosurfactant [10]. Environmental conditions studied include temperature, humidity, food level [11], agar rigidity [11, 73], oxygen availability [119], nearby interacting colonies [13] and the presence of antimicrobial agents [19], attractants and repellents [57].

## Statistical properties of the swarm dynamics

As detailed above, many of the biological studies on swarming bacteria describe in detail the biochemical manifestations of this phase [40, 60, 74, 75, 93, 129]. However, such studies provide limited information on the collective macroscopic properties of swarms that can consist of millions of cells. To this end, and at a risk of over-simplification, physicists tend to look at swarming cells as elongated (rod-shaped) self-propelled particles. The first quantitative physical-inspired works on collectively moving bacteria focused on swimming cells in sessile drops, or concentrated suspensions (e.g., [34, 119–121]). More recently, with better imaging abilities, swarming colonies were studied as well [6, 11, 12, 19, 33, 40, 142, 143]. However, the common denominator of all such examples is not only its phenomenological visible outcome of coherent swirling and dynamic clusters, but also the physics-motivated approaches and tools used to analyze them.

Most swarm experiments take place in a standard Petri-dish (8.8 cm in diameter) with a small (5-μl) drop of an overnight culture (typically containing ~ 10^6^ bacteria) inoculated in its center. The medium contains agar at different rigidities, from the softer substrates such as ~ 0.45% (agar concentration) for some species, up to much harder ones of ~ 2%. Nutrients vary depending on the species, but in many cases standard LB, peptone or some other yeast extracts and tryptone, are used. The plates are stored in an incubator with temperatures in the range of 25°-37 °C depending on the species.

**Fig. 2:**
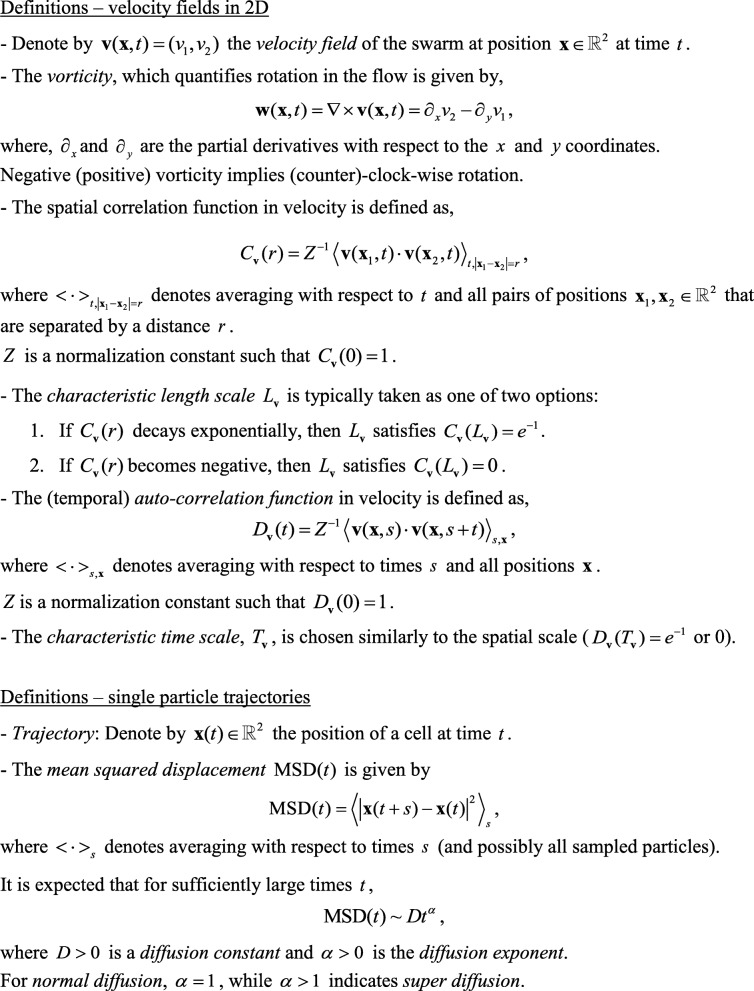
Definitions and formulae

The collective bacterial motion is typically analyzed using particle image velocimetry (PIV) algorithms (e.g., [143]) or optical flow (OF) (e.g., [103]). In principal, high-resolution (in time and space) movies are streamed into the computer, separated into frames, and after standard pre-processing for noise reduction and smoothing, the software identifies changing patterns in between consecutive frames. See an example in Fig. 1e. The denser the swarm, the more reliable will be the OF that does not track individual particles per-se. Such generic methods generate velocity fields, indicating the instantaneous motion of the flow in the observed field of view. Several derivatives of the flow fields are typically calculated, such as the vorticity field (the curl of velocity, or tendency of rotating), the distributions of velocities and vorticities, spatiotemporal correlation functions, indicating the characteristic length (the typical size of vortices in the flow) and time scales (the typical life-time of a vortex) of the dynamic flow. See Fig. 2 for precise definitions and below for experimental results.

Below, we distinguish between monolayer and multilayer swarms. The observed swarming dynamics strongly depends on several factors such as the species under study (or the specific strain), the nutrients levels, and the wetness (or humidity). Multilayer swarms are usually more localized as far as the expansion of the colony is concerned. These differences are also manifested in several characteristics of cells. For example, the same species is typically longer in monolayer swarms compared to thicker swarms. Because nature does not provide easy-to-find habitats for swarming, there is no single protocol for lab experiments and it is important to test different conditions.

### Multilayer swarms

#### Variations of agar concentrations and nutrients level

It is expected that the ability of bacteria to move effectively will depend on the available nutrients (energy source) and medium type. This motivated research to explore the dynamical properties of collective behavior under a variety of conditions, in particular adverse environments. In canonical conditions, namely when cells are not starved (peptone experiments), the substrate is moist, and humidity and temperature are favorable for the cells, *B. subtilis* may form multilayer swarm colonies. Macroscopically, the colony expands outwards with a circular symmetry and a constant speed (~ 1 μm/sec). On the microscopic scale, the distribution of velocities is Gaussian and the mean speed is about 25 μm/sec. The dynamic patterns that are formed yield spatiotemporal correlation functions that decay exponentially, both in time and space; hence, *B. subtilis* moving under canonical conditions obey normal statistics. This is also true for *P. dendritiformis* grown under canonical conditions [11], even if food levels and the rigidity of the surface (agar concentration) are dramatically varied. Manipulation of the substrate by addition of surfactants does not change these observations, although the colonial expansion speed and the microscopic speed may vary.

#### Addition of antibiotics

Increased resistance of swarming bacteria to antibiotics was linked specifically to swarming motility and not to other types of movement [76, 90]. In particular, it cannot be attributed to antibiotic-resistant mutations [78]. This raises the question of how antibiotics affect the physical properties of the swarming dynamics? Understanding these effects may shed light on the mechanisms underlying antibiotic resistance. For *B. subtilis*, when exposed to sublethal concentrations of kanamycin (which reduces the motility of affected cells), the collective dynamics transitions from normal to anomalous behavior, with a heavy-tailed velocity distribution and a two-scale temporal relaxation decay of the normalized velocity field [19]. It was found that this anomalous, non-Boltzmann dynamics is caused by the formation of a motility-defective subpopulation that self-segregates into clusters. This observation was verified both experimentally, using a mixture of motile and immotile *B. subtilis* cells, and theoretically, using simulations of a mixture of driven inelastic spheres. Interestingly, although the microscopic speed was dramatically reduced, the expansion rate of the colony edge, and the number of live cells extracted from the leading edge were not affected by kanamycin [19].

**Fig. 3:**
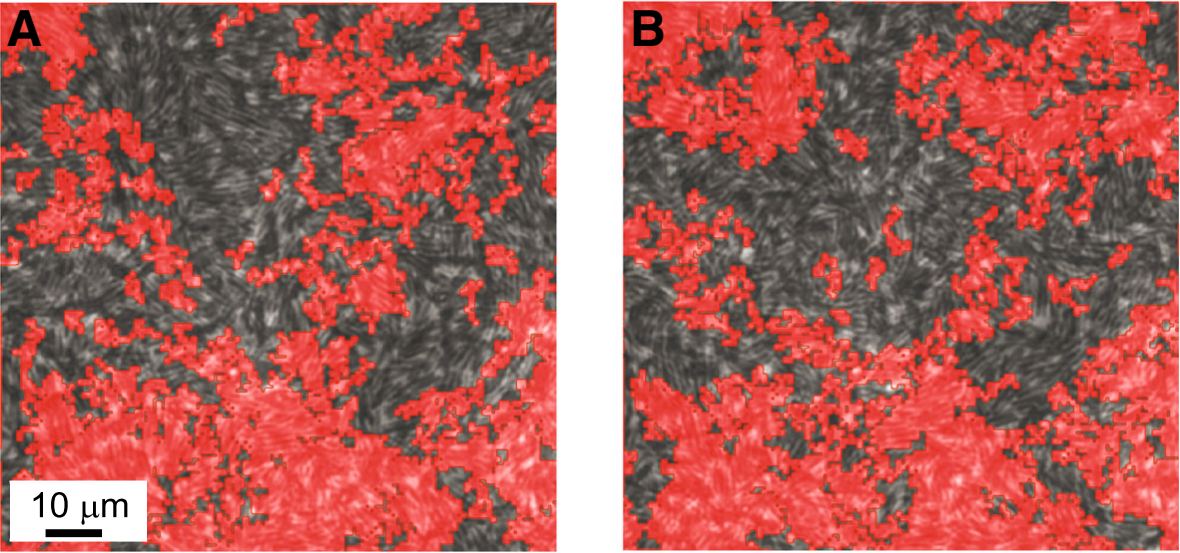
Antibiotics resistance – segregation into clusters. **a** and **b** are two images showing the same field of view, 1 s apart. Red regions are very-slowly moving cells corresponding to the motility defective bacteria. Segregation is relatively constant in time and space, so that the red regions remain in the same places

Why wasn’t the growth of the colony affected by antibiotics? The answer is that addition of kanamycin increases the fraction of immotile cells in the population. If motile and immotile cells were mixed, then the entire colony may become jammed [26, 138] and unable to grow. However, the system segregates into clusters of immotile cells, while the unaffected cells migrate freely. As a result, the expansion of the colony is not affected. The appearance of islands, corresponding to immotile, antibiotic affected bacteria, can be explained in terms of the physical properties of granular materials, as discussed below. In other words, the colony survives thanks to a physical phenomenon, rather than a biological one. In Fig. 3 we show an example for the segregation of immotile cells into clusters (the encircled red regions) that are relatively fixed, allowing the motile cells to migrate.

#### Chemotaxis, tumbling and rotational diffusion

Most swarming species can easily swim in liquid bulk. If the cells are sparse enough, interactions between individuals are negligible, and their motion has typically the form of run-and-tumble, characterized by straight trajectories (runs) interspersed by shorter, random reorientation (tumbles) [16, 127]. During runs, cells rotate their flagella in a counterclockwise direction, which creates a bundle that propels them forward. The duration of runs is approximately exponentially distributed, with an average of 1–2 s. However, the mean run-time is not constant, but can slowly change in time [18, 21]. During tumbles, the flagella rotate in a clockwise direction and the bundle opens; as a result, the cells randomly obtain a new direction in which a new run event takes place. The chemotaxis signaling network operates in controlling the duration of runs, enabling navigation towards or away from desired regions in the medium. In contrast, while in dense populations, flagellated bacteria exhibit collective motion and form large dynamic clusters, whirls, and jets, with intricate dynamics that is fundamentally different than trajectories of sparsely swimming cells. Although swarming cells do change direction at the level of the individual cell and may exhibit reversals [128], it has been suggested that chemotaxis does not play a role in multicellular colony expansion [24, 84]. Instead, changes in cell direction stems from flagellar rotor switching that are uncorrelated with the chemical cues [116].

One method for studying the role of tumbling on the swarm dynamics is by comparing wild type (WT) cells with mutants that do not tumble, or with mutants that tumble at random times – independently of the chemotaxis system. Smooth swimming bacteria are cells that do not tumble, either because these species simply do not tumble, or because they were genetically modified in the lab not to do so. These cells exhibit the run phase only, meaning that they swim in relatively straight trajectories. Almost all swarming species that were mutated to swim smoothly do not swarm at all, or they need some “assistance” to swarm on agar; for example, *S. marcescens*. If inoculated on agar, the growing colony expands much slower, and on the microscopic scale the cells do not show the characteristic whirls and jets, but some weak motion. Some “tricks” were found to assist the cells in swarming. These include spritzing of water on the colony to enhance wetness, initial inoculation of a much larger volume compared to the regular case (e.g., placing 100-μl of overnight culture instead of 5-μl), or using fresh swarm plates i.e., plates that are highly moist in the first place. In all cases the swarm is still different compared to WT colonies.

In some studies, it was suggested that the role of tumbling, or perhaps the role of rotor switching between the run and the tumble, during motion on agar or swarming has additional functionality, such as pumping liquid from the agar [128] or stripping off lipopolysaccharide (LPS) from the Gram-negative outer membrane to enable wetting of the surface [124, 134]. In the absence of tumbling or rotor switching, the local wettability is poor and the cells are stuck. Therefore, in order to test the role of tumbling, it is important to disconnect the chemosensory system from the rotor switching, and test mutants that do tumble but do not have a functional chemosensory system.

In *S. marcescens*, the smooth swimming mutants show a very poor swarming pattern with no motion at the edges, and robust motion only in the interior of the colony. To the naked eye, the colony seems to swarm because it expands rapidly, but the microscopic picture shows the poor motion at the edges. In contrast to smooth swimming mutants, *S. marcescens* can also be mutated to form spontaneous tumbling. These cells, which tumble at random times that are independent of the chemotaxis system, were found to swarm very similarly to the WT. In *B. subtilis*, smooth swimming mutants do swarm. To the naked eye, their motion looks similar to WT swarming. In addition, spatiotemporal correlation functions as well as the distribution of velocities is similar [116]. However, a correlation between the velocity and the vorticity was obtained for the mutated strain and the ability of single cells embedded in a swarm to follow the crowd is different (see more results on motion of individuals in “An individual within the crowd” Section). On the other hand, non-chemotactic cells that do tumble exhibit the same behavior as the WT. This demonstrates that chemotaxis per-se does not function during swarming even though that tumbling, or rotor switching, does play a role during swarming.

As will be discussed below, the subtle differences in the flow statistics of WT and smooth-swimming cells results in slightly different geometrical properties of cell trajectories. In particular, WT cells have a slightly larger diffusive exponent [116]. This means that the displacements of a WT cell (with rotor switching) in a swarm are (on average) slightly larger than that of a smooth-swimmer. This is counter-intuitive as one would expect that tumbling would lower super-diffusion. Once again, we see that the biological properties of cells (in this case rotor-switching) affect the physics of the system and the environment each cell senses.

#### Aspect ratio

The theory of active matter, in particular, in relation to self-propelled rod-shaped particles, predicts that the shape of cells should play a central role in determining the flow pattern and its statistics. This is due to the fact that both excluded volume effects and hydrodynamic interactions produce an effective alignment mechanism that depends on the cell aspect-ratio [3, 97, 135, 137]. Quantitative statistical studies of collectively swimming bacteria, and on gliding ones [136], were performed prior to flagellated swarming (e.g., [3, 34, 45, 120]). In theoretical studies that model bacteria as self-propelled rods, particles with small aspect ratios formed tightly packed clusters that prohibited the formation of swarming (a jammed state) [135, 137, 138]. For longer rods, swarming (a non-jammed state) was obtained at low densities, “delaying” the jammed state to much higher densities.

**Fig. 4:**
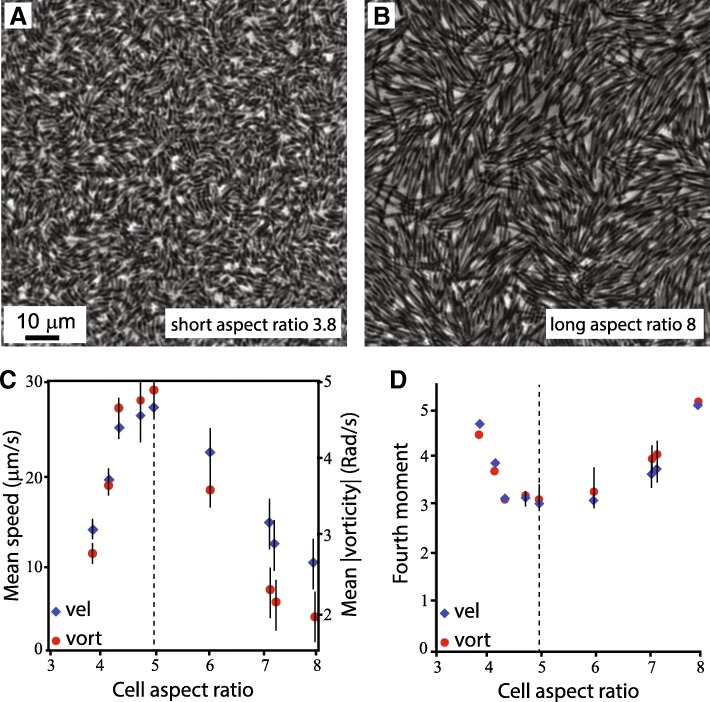
Effect of cell aspect ratio. **a** and **b** are top-view images of the swarm for short (aspect ratio = 3.8) and long (aspect ratio = 8) cells. **c** The mean microscopic speed of the cells depends on the aspect ratio in a non-monotonic way. Cells that are close to the WT in aspect ratio exhibit faster speeds (and vorticity). **d** The kurtosis (indicating the deviation from Gaussian) of the distribution of velocities and vorticities exhibit a non-monotonic trend with a minimum for the WT and strains with similar aspect ratios

To test the role of cell aspect ratio on bacterial swarming, several variants of *B. subtilis* differing only in aspect ratios were compared ([65]; for more details about strains see [55, 88, 95]). Figures 4a-b show an example of the microscopic view of short and long cells. In general, all strains formed a swarm pattern, and the changes between the cases were measured. Firstly, the average microscopic speed was found to depend on the aspect ratio in a non-monotonic way, with slower motion for colonies composed of short and long strains, and faster motion for the WT colonies (Fig. 4c). Moreover, the velocity of both shorter and longer cells has an anomalous, non-Gaussian, distribution. Figure 4d depicts the scaled 4th moment (kurtosis), *κ* = *M*_4_/*σ*^4^, where *M*_4_ is the centered fourth moment and σ^2^ the variance. While WT cells have a kurtosis of approximately 3 (Gaussian), both long and short cells show a higher kurtosis, which can be up to 5, indicating a heavier tail. Similar results are obtained for the distribution of the vorticity and the temporal correlation function (e.g., Fig. 3 in [65]).

These experiments show a significant, qualitative dependence of the swarm dynamics on the aspect ratio. In particular, it appears that WT, or mutant cells of similar aspect ratios, are optimal in this sense. This result is particularly striking given the fact that some bacterial species change their cell aspect ratio before starting to swarm, suggesting that the aspect ratio (typically about 5:1) is important to the swarming dynamics.

### Monolayer swarms

**Fig. 5:**
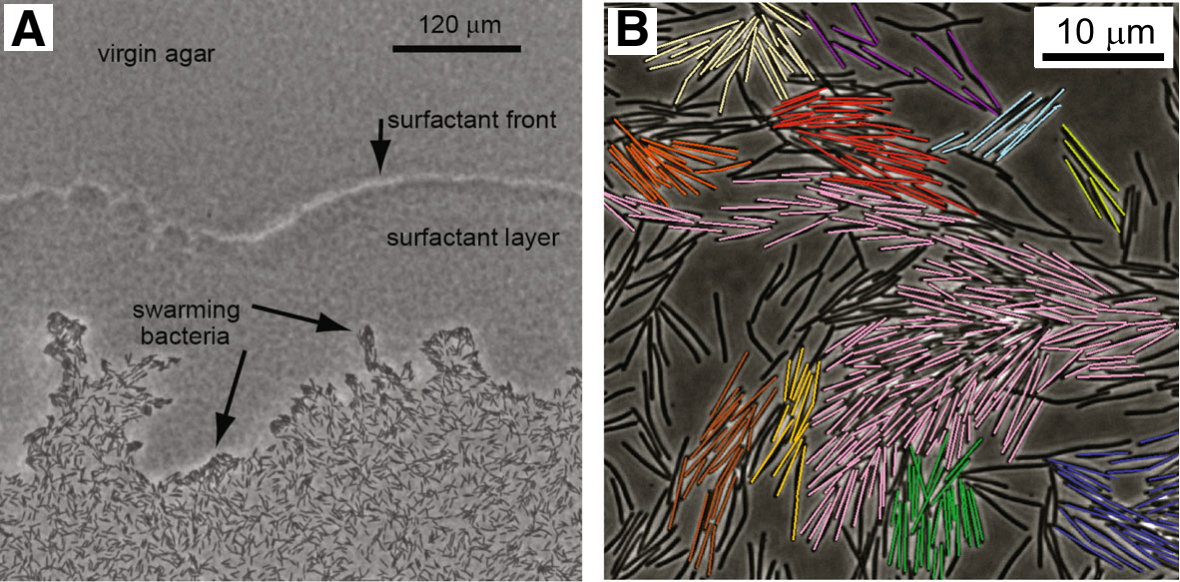
Swarming in a monolayer. **a** The cells are sparse and move on the agar in a single layer. A surfactant layer secreted by the cells is obtained ahead of the colony. **b** A larger view of a monolayer swarm. Cells move in dynamic clusters that split and merge. Colors indicate cells that “belong” to different clusters

The previous subsection described results for swarms which are thin, yet include a few layers of cells. In some cases (e.g., *B. subtilis* grown on LB plates), swarming bacteria may form fast-expanding colonies, where the cells cover the Petri-dish in a few hours [10, 33, 60, 142]. These cases are typical for surfactant secreting cells, where the surfactant rapidly spreads ahead of the colony reducing the surface tension of the liquid in which the bacteria move, enabling a much faster migration (Fig. 5a). Due to the reduction in surface tension, the water captured by the swarm collapses and cells are sparse; they move in small clusters near the colony edge, but form larger clusters in the interior. In general, this type of swarming yields a monolayer of bacteria with a covering surface fraction of moving cells between ~ 0.15 and ~ 0.85, depending on the distance from the edge. The number of studies on monolayer swarming is relatively small.

The denser the cells, the faster they move. This is the hallmark of collective motion – showing that many cell cooperate to produce faster motion. In addition, the denser the cells, the larger is the variation in local instantaneous density; this is due to the fact that dynamic clusters split and merge which eventually leads to giant number fluctuations in density (see Fig. 5b and below for details) [142]. Defining clusters and obtaining the distribution of cluster sizes can be done in different ways. For example, Chen et al. [33] and Zhang et al. [142] define two cells to belong to the same cluster if the distance between their centers and the differences in orientations are smaller than given thresholds. It was shown that the distribution of cluster sizes decreases exponentially with the cluster diameter ([33], Fig. 2b). However, the distribution of the number of cells within a cluster is more complicated and fits well a power law with an exponential cutoff ([33], Fig. S5 and [142], Fig. 4). The dependence of the cluster length on the number of bacteria is not clear [33]. Fluctuations in speed, direction of motion and spatial correlations exhibited long-range correlations [33]. More precisely, it was argued that the correlation length is proportional to the size of the cluster. These results are in line with recent theories suggesting that long-range scale-invariant correlations may be a general feature in systems exhibiting collective motion [105]. However, because of the particular method used to define clusters (ordered regions have, by definition, larger clusters), we find these results regarding swarming bacteria problematic.^1^

## Theoretical aspects

In this section, we briefly review some of the relevant theoretical aspects underlying the statistical physics of bacterial swarming. This topic sits at the intersection of several current active areas of research (1) Collective motion in nature, (2) The physics of micro-swimmers and (3) Active matter – in particular the statistical physics of self-propelled elongated particles. These topics are, of course, related and overlapping. Below, we briefly describe each one with an emphasis on the main progress that is relevant to bacterial swarming.

### Collective motion

It has long been recognized that many natural phenomena that involve collectively moving organisms – from cells to mammals, share some similarities and universal features [37, 51, 123, 126, 132]. The modern theoretical study of these systems started with the phenomenological models of Aoki [2] and Reynolds [110] who were interested in generating motion patterns that appear similar to those of fish or birds. The physical point of view has largely grown from the pioneering work of Vicsek et al. [131], who studied models of self-propelled agents with alignment interactions and noise, and the continuous approach of Toner and Tu [125, 126]. Those approaches, as well as the many later suggested models and variations (e.g., [30, 31, 52–54, 99, 112]) showed that such systems undergo a phase transition between a disordered phase at low densities (or high noise) and an ordered phase at high densities (low noise). The transition explains how local (short-range) alignment between animals can propagate throughout macroscopic swarms to produce large-scale order and synchronization. The stability of the ordered (or disordered) states has also been analyzed rigorously in even more simplified models (e.g. [1, 8, 20, 28, 39, 42, 56, 100]). For recent reviews on the theory of collective motion see [132]. Broadly speaking, “general-purpose” models of collective behavior fall short of providing a good description of the phenomena of collectively swimming bacteria in general and bacterial swarming in particular.

### Micro-swimmers

Physically, fluid flow is characterized by the Reynolds number [101], which is a dimensionless number that (loosely speaking) quantifies how turbulent is the flow (a high Reynolds number implies more turbulence). Given their small size and the medium in which they swim (and swarm), bacteria move in a highly viscous environment [47]. For comparison, the Reynolds number of a small fish in the ocean is around 10, while for swarming bacteria it can be as small as 10^− 5^. This implies that acceleration is negligible, which means that if cells cease rotating their flagella, they stop instantly. The study of swimming at low Reynolds number, including by organisms, was initiated by Purcell about 40 years ago [102]. Current theories yield highly accurate approximations of the hydrodynamic flow a cell generates in 3D fluid. In particular, it has been shown that this flow creates an effective aligning force between cells. For a recent review see [47].

One simple way to approximate the flow is through a multipole expansion. The rotating motion generates a force (called a force monopole), which pushes the cell forward (and the fluid backward). However, as the cell-body is pushed through the fluid, it creates a counter-acting force with the same magnitude but in the opposite direction. Since the cell body is slightly displaced from the hydrodynamic center of the flagella, they do not cancel each other exactly but form a flow dipole [47]. Assuming Stokes-flow (Reynolds number = 0), the expression for the flow can be obtained analytically [101]. This method and similar ones have been used as the basis of simulations that study the physical properties of dilute to moderately dense particles (typically with periodic boundary conditions). Excluded volume interactions are also typically taken into account [108]. Both three-dimensional (3D) and quasi-2D systems were studied [7, 108]. The main result of such simulations is that an ordered phase, in which all particles are oriented in the same direction, becomes unstable at high densities [34, 52, 113, 117].

### Active matter

Active matter refers to particles or organisms (agents) that have their own source of energy that is typically used to generate movement. As a result, these systems are inherently out-of-equilibrium [83, 104]. Examples range from vibrated particles [23, 44, 77, 89, 105], swimming sperm [111], Janus particles [26] to moving animals [51], including, of course, bacteria [145]. While researches have been interested in such systems for decades [49], a unified view of active matter is a relatively recent approach [83] and the literature is growing rapidly. We will focus on a few main features, which have been identified to be the hallmark of self-propelled matter.

For systems at equilibrium, it is expected that fluctuations in measuring the density should decrease like the square root of the sample size. The reason is that the density *ρ* is the mean number of particles *N* counted in a given area *A*, *ρ* = *N*/*A*. The central limit theorem, which holds at equilibrium (unless the system is close to a continuous phase transition), implies that the standard deviation in the number of cells observed, Δ*N*, is proportional to $\sqrt{N}$. This implies that Δ*ρ*/*ρ* = Δ*N*/*N* ∼ 1/$\sqrt{N}$, which decreases to zero with density or sample size. In contrast, theoretical models showed that for active systems, which are not at equilibrium, the central limit theorem fails. It has been predicted that in active systems Δ*N* will be proportional to *N* [86, 96, 105, 114, 115, 117]. This implies that density measurements should be highly irregular. The phenomenon, referred to as giant number fluctuations, has been observed experimentally in non-living systems of active matter [44, 89, 91]. Zhang et al. [142], showed that swarming bacteria indeed show giant number fluctuations, although with a slightly lower exponent (Δ*N* ∼ *N*^0.75^). This result is a clear indication that swarming bacteria constitute a natural example of collective motion.^2^

A second prediction of active-matter theories are heavy-tailed auto-correlation functions in the single-particle velocity [105, 117]. The name heavy-tail typically implies that *D*_***v***_(*t*) decays at large time as a power-law, *D*_***v***_(*t*)~*t*^−*α*^. See Fig. 2 for definitions. With 0 < *α* ≤ 1 the integral over time (from 0 to infinity) diverges and the system is said to have infinite correlations. In [105], it is predicted that in 2D active systems *α* = 1. Measuring the velocity correlation function of individual swarming cells is challenging, since it required tracking a single cell that it is moving amid a dense colony for long times. Recently, these heavy time-tails have been confirmed experimentally as described below (“An individual within the crowd” section).

The last property of active system we review are order instabilities at high densities and wave-lengths [34, 117]. It has been suggested that global alignment of polar self-propelled particles (i.e., particles that have a direction and align it with their neighbors) is unstable [52, 113, 117]. This has been predicted both in continuous and agent based simulations [117], as well as analytically [1, 8, 20, 100, 125]. In other words, if one could somehow arrange all bacteria to move in exactly the same direction, then this order will quickly break due to fluctuations. The manifestation of this property in bacteria swarming gives rise to an intermediate length scale (larger than single bacteria but smaller than the swarm) on which the bacterial rotational motion occurs [46, 139]. Termed meso-scale bacterial turbulence, the size of these vortices can be approximated based on first principles, i.e., given the physical properties of cells and the medium (size, aspect ratio, density, viscosity etc.) [34, 63].

### Bacteria

Swarming bacteria are a quintessential example of active micro-swimmers that are moving collectively. As a result, a theory of bacterial swarming, which is far from complete, borrows heavily from all three disciplines. The bacterial cell, together with the many flagella surrounding it, give rise to inelastic cell-to-cell collisions. Together with the elongated shape of swarming cells, collisions are a source of short-range alignment [34, 52, 97, 138]. In addition, the hydrodynamic flow generated by the rotating flagella (approximately a dipole) is a source of long-range alignment [3, 9]. Overall, swarms are well approximated by such models, either agent-based or continuous [3, 9, 34, 46, 97, 137]. Indeed, we have seen that bacterial swarms show much of the predicted physical phenomena such as instabilities in long-range polar (and nematic) order [85] and giant number density fluctuations [98, 142]. As explained above, the observation of long-range order (or scale-invariant correlations) is not fully resolved. Even more so, all experiments to date point out that the dependence of all the measured dynamical quantities on density is continuous [34, 46, 120, 122, 137]. Recently, Jeckel et al. [68] studied the available phases in an expanding swarming colony of *B. subtilis* and discovered regions corresponding to different dynamical properties. However, to date, no phase transitions (in the sense of abrupt shifts between quantitatively distinct dynamical states as a function of some controlled parameters) were experimentally identified in bacterial swarms.

Models for swimming bacteria have been successfully applied to describe many aspects of the observed dynamics, including the increasing speed dependence on concentration [34], the decay of correlation functions (for WT cells) [34, 107], reduction of viscosity [35, 50, 81, 121] and the appearance of meso-scale turbulence [137, 138]. However, despite considerable successes, the physics described above ignores many of the features that were experimentally found to be essential to our understanding of the collective flow, such as the role of rotor switching or tumbling [116], and the anomalous statistics observed with short and long cells [65].

## An individual within the crowd

So far, we have referred to the swarm as if it were a group phenomenon. Cells move collectively, form distinct flow patterns and migrate together. Do all cells in the swarm do the same? Are some cells able to move in directions that are different from their close neighbors? What is the role of propulsion of a single cell in the swarm? Can it “decide” on its direction or does it simply go with the flow? Such questions are important both from the physical side, concerning forces between the individual and the group, but also biologically, concerning the ability of individuals to disperse and disseminate their DNA.

Tracking individuals within a swarm is not an easy task. In Turner et al. [127, 128], individual *E. coli* cells were labelled, first in liquid [127] and then within a swarm [128], using different fluorescent techniques, including labelling the cell body and the flagella. The results they have obtained were the first to lay the basis for tracking techniques in dense bacterial swarms. For instance, they have shown that a single cell exhibits different modes of motion, which are related to rotor reversals. In Tuson et al. [129], working on *P. mirabilis*, the flagella of bacteria in a swarm was labelled. This study looked at how the bacterial density regulates motility in viscous environments, and reviled details about the interaction between flagella in *E. coli* [38].

In most Gram-positive species, labelling the cells, all the more their flagella (or sometimes simply turning on the fluorescent light), destroys cell motility. The reason why this happens is not completely clear. However, recent methods yielded intense and bright labelling of *B. subtilis* with no photobleaching, and zero influence on the motility from the light source (e.g., [5]). Recently, successful labelling of flagella in some strains of *B. subtilis* was also achieved, showing how they operate during motion on a surface [79].

### Lévy walks

**Fig. 6:**
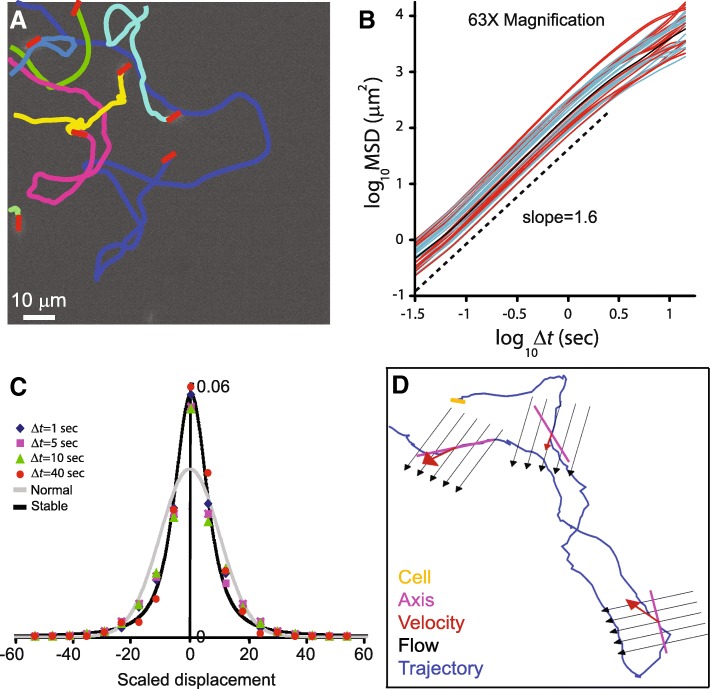
Individual cells within a dense swarm. **a** Trajectories of fluorescently labelled cells. **b** The mean-squared displacement shows super-diffusive behavior. Each line shows the statistics obtained for a single cell. **c** Following proper scaling, the displacement of positions of cells (along the *x* or *y* axes) collapse on a master curve which is approximately a Lévy stable law. **d** A trajectory of a single bacterium showing the instantaneous cell-orientation, velocity and the flow around it

By using some of the above-mentioned labelling techniques, high-resolution imaging and tracking algorithms, the precise trajectories of individual bacteria, moving among their many siblings in a swarm, was achieved (Fig. 6a-c). Multilayer swarm colonies, of both *B. subtilis* and *S. marcescens* (separate experiment for each of the species), were grown from a mix of WT cells and fluorescently labeled ones, at a ratio of 1000:1. By tracking the trajectories of the fluorescently labelled individuals, it was found that the bacteria are performing super-diffusion, consistent with Lévy walks [141]. Lévy walks, which are characterized by trajectories that have straight stretches for extended lengths whose variance is infinite, has been reported on a large variety of organisms and particles – from T-cells to humans [109, 133]. However, the evidence of super-diffusion consistent with Lévy walks in bacteria suggests that this strategy may have evolved considerably earlier than previously thought.

Single bacterial trajectories were analyzed statistically in order to obtain their characteristics. First, a mean square displacement (MSD) plot was obtained with a slope *α* = 1.6, indicating super-diffusion (Fig. 6b). See Fig. 2 for definitions. A number of statistical tools and tests have been suggested in order to identify Lévy walks [5, 59]. For example, one of the hallmarks of Lévy walks is that the distribution of displacements, i.e. the histogram showing the displacement of cells within a given time Δ*t*, is expected to be a symmetric Lévy stable distribution with parameter 3-*α* (Fig. 6c).

The key to understanding and predicting many phenomena lies with the identification of an underlying generative mechanism. In [4], a new mechanism for Lévy walks, explaining the recently observed super-diffusion of swarming bacteria has been suggested. The model hinges on several of the key physical properties of bacteria which were described above, such as an elongated cell shape and self-propulsion. The model described the motion of a single cell as it pushes itself within the effective (and greatly simplified) vortex-like flow generated by the swarm. It was shown that trajectories of cells are chaotic. This chaos yields erratic trajectories whose geometric properties resemble that of swarming bacteria. Biologically, the model shows that the properties of the bacterial trajectories are plastic, i.e., they can be tuned by adjusting the mechanical properties of cells, for example, speed of self-propulsion or aspect-ratio. This idea is consistent with the observation that indeed bacteria change their cellular properties prior to swarming.

### Swarming cells move against the flow

Now that the trajectories of the individuals were analyzed, it is important to see how these cells move in respect to the flow that exhibits normal statistics. To this end, two fields of view were captured simultaneously: one for the entire swarm and one for the fluorescent cells, which are the same as their siblings, but differ in the fact that they glow. In this way, one can superimpose the instantaneous motion of the individual with respect to the instantaneous velocity field around it [107].

Figure 6d shows a cartoon of the flow-field obtained from the optical flow superimposed with an example individual trajectory and the cell orientation. To “calibrate” the accuracy of the measure, immotile fluorescent cells were also embedded in active WT non-labelled cells; these immotile cells are unable to generate motion, and their trajectories are only due to the motion of the crowd. By looking at the differences between the angles of the flow, direction of cell motion and alignment of the cell, one can see that the probability of finding large angles is very small, indicating that the immotile cells approximately follow the flow. However, the fluorescently labelled motile cells, embedded in the swarm, may move in directions much different than the flow, or may be miss-aligned with the velocity field [33, 107]. For similar results in swimming bacteria, see also [82, 120].

## Further swarming related phenomena

### Back and forth motion and curly patterns

**Fig. 7:**
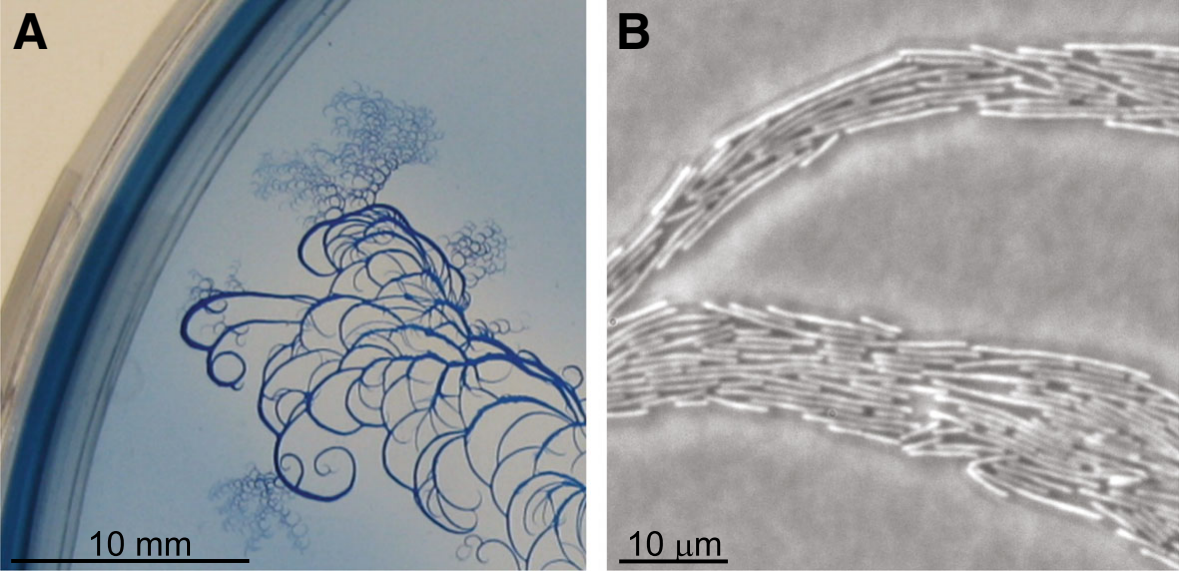
Chiral movement of swarms. **a** A macroscopic view of the curls formed when *P. dendritiformis* type-*C* grow on agar. No particular curvature is obtained, but the direction is the same. **b** The microscopic view of a colony similar to the one seen in (**a**). The “roads” of bacteria are thin curls. The elongated rod-shaped cells move along these roads back and forth

Bacterial cells of the species *P. dendritiformis* type-*C* are elongated rods, with an average aspect ratio of 17 ± 12 μm. These bacteria move back and forth on the agar, changing direction of motion every ~ 30 s, creating “roads” of cells with no typical thickness - from 1 μm (composed of few moving cells) to 1 mm (thousands) [12]. Whether the cell length is the reason for their unique motion, or otherwise they also form a swarm structure much different than shorter cells such as *P. dendritiformis* type-*T*, *B. subtilis* or *E. coli* – the macroscopic shape of the colony has a structure of curls which share the same chirality. See Fig. 7 and [14] for experiments and models.

### Thick swarms and motion along the *z*-axis

Some bacteria such as *E. coli*, *Salmonella* and *S. marcescens* form very thick swarm colonies (~ 40 μm in thickness), in particular at the inner colony regions, e.g., few 100’s of microns towards the interior of the colony. In *S. marcescens*, based on off-focus fluorescent imaging, the three-dimensional dynamics and geometry of the swarm was studied [92]. It was shown that the cells rigorously swarm in three dimensions and inhabit mostly the middle “floors” of the colony. While cells do get to the bottom of the colony, they rarely migrate to the upper parts of the colony, which contain mostly liquid. It was found that secreted biosurfactants keep bacteria away from the swarm-air upper boundary, and that added antibiotics at the lower swarm-surface boundary lead to their migration away from this boundary. Formation of the antibiotic-avoidance zone is dependent on a functional chemotaxis signaling system, in the absence of which the swarm loses its high tolerance to the antibiotics. Once again, we see that the biological properties of cells (repulsive chemotaxis) affects the physics of the system and the environment each cell senses.

### Swarming bacteria transport materials

One of the striking observations in swarming bacterial colonies is that swarms can carry (or transport) materials, such as small beads [10] or even other organisms. It has been shown that *P. vortex* can transport micro-meter beads, other bacteria [48] or even algae [66] in order to bypass obstacles, increase antibiotic resistance or facilitate faster colony growth [22]. While the precise mechanism in which *P. vortex* and its “cargo” attach is unknown, it has been suggested that the flagella entangles with the transported object, creating an effective drag [118].

### Non-flagellated swarmers

Some species, such as myxobacteria [72, 136, 140, 144], migrate collectively on surfaces using motive organelles different than flagella. These include, for example, motors that are based on pili, and gliding (slime). Studies on myxobateria revealed that periodic reversals in their motion on agar allow them to spread efficiently, and that mutants lacking some genes migrate poorly [140]. Other studies demonstrated, experimentally and theoretically, the role of a biochemical signaling system where intracellular dynamics, contact-mediated intercellular communication, and cell motility all lead to group behavior producing collective motion and intricate periodic patterns in a form of waves [64, 69, 136]. Theoretical myxobacteria-related studies include discrete [69, 97, 98] and continuum [27] models, each suggesting different types of interactions between the individuals among the group.

### Swarming throughout an expending colony

In [68], Jeckel et al. used a wide range of statistical observables to quantify the swarming dynamic at different regions of an expending colony of *B. subtilis*. Using machine learning clustering techniques, they identified 3 distinct dynamical states: a low-density single-cell phase; a high-density rafting phase with a high percentage of comoving cells and a biofilm phase characterized by long, unseparated cells. Two coexistence phases were also observed.

## Conclusion, summary and outlook

Bacterial swarms are a fascinating natural system exhibiting collective motion in which millions of cells participate in generating complex motion patterns. One of the main reasons for the difficulties in deciphering the basic principles underlying the swarm formation and its dynamics is that both physics and biology play a pivotal role. It is clear that cells obey the laws of physics and are constrained by the physical principles governing collectively moving dense suspensions of active micro-swimmers. In this review, we tried to highlight a complementary approach, suggesting that physics pose not only constraints, but also an opportunity for the cell. Under harsh conditions, bacteria develop sophisticated survival mechanisms. In order to flourish and invade new territories, bacteria may have evolved to manipulate the cellular mechanical properties as well as the physical properties of their medium, in order to create advantageous dynamics. In this sense, the biology of swarming bacteria promotes favorable physics to aid in their survival.

Despite considerable progress, much of the physical principles underlying swarms are still far from fully understood. For example, the theory of collective motion predicts a phase transition between a disordered phase at low densities and an ordered one at higher densities. Such a transition has not been observed experimentally in collectively moving bacteria. One reason that phase transitions were not observed is the technical difficulty in manipulating some of the fundamental global properties of the swarm such as control of the density and aspect ratio.

It is expected that a natural habitat for swarms will typically include several species proliferating and moving in complicated, heterogeneous environments. In such mixed colonies, species may differ in both their biological (e.g., expressed genes, secreted toxins) and physical properties (e.g., size, propulsion mechanism, surfactant production) move and cooperate in order to enhance each species survival, while at the same time, compete over available resources [15]. Understanding the physics of mixed systems takes the next step towards understanding how real and natural bacterial swarms behave.

## Abbreviations

2D: Two-dimensional
3D: Three-dimensional
LPS: Lipopolysaccharide
MSD: Mean square displacement
OF: Optical flow
PIV: Particle image velocimetry
WT: Wild type

## Footnotes

1 The claim that the average cluster size of ordered particles is larger than the size of disordered clusters is problematic because of the method Chen et al. [33] define clusters: two cells are said to belong to the same cluster if the distance between the cell centers and the differences in orientations are smaller than given thresholds. Since the minimal distance is fixed for all measured densities and is smaller than the typical cell length (but much larger than the width), it is expected that the width of clusters (in the direction perpendicular to the mean cluster orientation) will be proportional to density, while the length of clusters is (approximately) independent of density (because cells cannot crowd in this direction). Indeed, Fig. 2a of [33] appears linear after removing the first two points (corresponding to the smallest clusters). See also ([142], Figs. 2a-b) which compares the pair correlation function at two densities. As a result, the number of cells in a cluster is expected to be proportional to the overall density ([33], Fig. 2b). This observation is a result of the way clusters were defined and may not be of particular physical or biological nature. A similar problem occurs in the finding that correlations are scale-free, i.e., that correlation lengths scale with the cluster size. Because clusters are defined as (close) cells that have similar orientations, large clusters are, by definition, more aligned (or the other way around – ensembles that are more aligned will be classified as in larger clusters). As the direction of the velocity is correlated with the direction of the cell body, it is expected that large clusters will show larger correlation lengths. In other words, the scale free property may be an artifact of the way clusters were defined.

2 The occurrence of large number fluctuations is related to what is termed motility-induced phase separation [29, 41, 87] in which cells (or particles) with reduced motility self-separate to form large immotile-clusters. This theory is not relevant for swarming bacteria because [29] assume that dense regions move slower – the opposite than observed with bacteria. In bacterial swarms, dense clusters move faster.
